# Supplementary material for: UK alcohol consumption during the COVID-19 pandemic: The role of drinking motives, employment and subjective mental health
Source: PLoS One. 2023 Apr 12;18(4):e0283233. doi: 10.1371/journal.pone.0283233 (PMC10096461; doi:10.1371/journal.pone.0283233)
Supplement: S1 File — (DOCX) [file pone.0283233.s001.docx]

**Supplementary Materials**

***Table S1. Parameters for initial model (2020 data)***

| **Regression Weights** | |  | Estimate | S.E. | *p* | Standardised Estimate |
| --- | --- | --- | --- | --- | --- | --- |
| employment location | <--- | Social Grade | -.651 | .031 | *** | -.317 |
| Mental health | <--- | Social Grade | .056 | .033 | .084 | .027 |
| employment security | <--- | Social Grade | .124 | .033 | *** | .061 |
| employment location | <--- | Gender | -.124 | .031 | *** | -.071 |
| Mental health | <--- | Gender | .297 | .032 | *** | .148 |
| employment security | <--- | Gender | .041 | .033 | .211 | .020 |
| employment security | <--- | Age | .003 | .001 | .009 | .043 |
| Mental health | <--- | Age | -.011 | .001 | *** | -.139 |
| employment location | <--- | Age | .007 | .001 | *** | .095 |
| Enhancement | <--- | employment location | -.048 | .017 | .006 | -.045 |
| Social | <--- | employment location | -.038 | .018 | .033 | -.035 |
| Conformity | <--- | employment location | .003 | .011 | .816 | .004 |
| Coping | <--- | employment location | .005 | .013 | .681 | .006 |
| Coping | <--- | employment security | -.102 | .013 | *** | -.121 |
| Conformity | <--- | employment security | -.063 | .011 | *** | -.092 |
| Social | <--- | employment security | -.017 | .016 | .275 | -.016 |
| Enhancement | <--- | employment security | -.017 | .016 | .275 | -.016 |
| Coping | <--- | Mental health | .187 | .013 | *** | .221 |
| Conformity | <--- | Mental health | .050 | .011 | *** | .074 |
| Social | <--- | Mental health | .049 | .018 | .006 | .045 |
| Enhancement | <--- | Mental health | .046 | .017 | .008 | .043 |
| AUDIT-C | <--- | Enhancement | .964 | .049 | *** | .371 |
| AUDIT-C | <--- | Social | .208 | .050 | *** | .082 |
| AUDIT-C | <--- | Conformity | -.387 | .064 | *** | -.096 |
| AUDIT-C | <--- | Coping | .711 | .051 | *** | .217 |
| **Covariances** |  |  |  |  |  |  |
| Gender | <--> | Age | -.536 | .104 | *** | -.084 |
| err13 (Enhancement) | <--> | err11 (Coping) | .358 | .015 | *** | .410 |
| err10 (Conformity) | <--> | err11 (Coping) | .176 | .010 | *** | .315 |
| err9 (Social) | <--> | err10 (Conformity) | .321 | .013 | *** | .435 |
| err13 (Enhancement) | <--> | err9 (Social) | .745 | .022 | *** | .646 |
| err13 (Enhancement) | <--> | err10 (Conformity) | .194 | .012 | *** | .268 |
| err9 (Social) | <--> | err11 (Coping) | .290 | .015 | *** | .326 |
| **Variances** |  |  |  |  |  |  |
| Social Grade |  |  | .238 | .005 | *** |  |
| Gender |  |  | .250 | .006 | *** |  |
| Age |  |  | 162.657 | 3.745 | *** |  |
| err14 (employment location ) |  |  | .885 | .020 | *** |  |
| err15 (Mental health) |  |  | .954 | .022 | *** |  |
| err16(employment security) |  |  | .994 | .023 | *** |  |
| err13 (Enhancement) |  |  | 1.133 | .026 | *** |  |
| err9 (Social) |  |  | 1.176 | .027 | *** |  |
| err10 (Conformity) |  |  | .462 | .011 | *** |  |
| err11(Coping) |  |  | .674 | .016 | *** |  |
| err12 (AUDIT-C) |  |  | 5.564 | .128 | *** |  |
| **Squared Multiple Correlations** | | |  |  |  |  |
| Mental health |  |  | .045 |  |  |  |
| employment security |  |  | .006 |  |  |  |
| employment location |  |  | .116 |  |  |  |
| Coping |  |  | .063 |  |  |  |
| Conformity |  |  | .014 |  |  |  |
| Social |  |  | .004 |  |  |  |
| Enhancement |  |  | .004 |  |  |  |
| AUDIT-C |  |  | .277 |  |  |  |

****p* < .001

***Table S2. Model fit statistics (2020 data)***

|  | DF | χ^2^ | *p* | NFI | CFI | RMSEA (CI) |
| --- | --- | --- | --- | --- | --- | --- |
| Initial model | 24 | 21.78 | < .001 | .920 | .923 | .074 (.069, .080) |
| Model 2 | 28 | 18.77 | < .001 | .919 | .923 | .069 (.064, .074) |
| Final model | 15 | 1.63 | .059 | .996 | .999 | .013 (.000, .022) |

***Table S3. Indirect effect values (2020 data)***

| **Indirect Path** | **Estimate** | **Lower** | **Upper** | ***p-*value** | **Standardised estimate** |
| --- | --- | --- | --- | --- | --- |
| employment location --> Enhancement --> AUDIT-C | -.039 | -.064 | -.014 | .006 | -.014** |
| employment location --> Social --> AUDIT-C | -.007 | -.014 | -.001 | .044 | -.002* |
| employment security--> Coping --> AUDIT-C | -.072 | -.091 | -.055 | .001 | -.026*** |
| employment security--> Conformity --> AUDIT-C | .021 | .014 | .031 | .001 | .008*** |
| Mental health --> Coping --> AUDIT-C | .136 | .114 | .164 | .001 | .049*** |
| Mental health --> Conformity --> AUDIT-C | -.017 | -.028 | -.01 | .001 | -.006*** |

**p* < .05, ***p* < .01, *** *p* < .001

***Table S4.Model parameters for M1 (Baseline model) 2018 vs 2020 data***

| 2018 | Unstandardised estimate (SE) | z-value | *p*-value | Standardised estimate |
| --- | --- | --- | --- | --- |
| Gender | -1.066 (.052) | -20.484 | *** | -.190 |
| Age Group | -.075 (.017) | 4.406 | *** | .042 |
| Social Grade | -.308 (.053) | -5.836 | *** | -.054 |
| Enhancement | 1.061 (.034) | 31.258 | *** | .405 |
| Social | .224 (.033) | 6.862 | *** | .088 |
| Conformity | -.592 (.045) | -13.091 | *** | -.139 |
| Coping | .637 (.035) | 18.284 | *** | .197 |
|  |  |  |  |  |
| AUDIT_C |  |  |  |  |
| Intercept | 3.207 (.162) | 19.772 | *** | 1.142 |
| Variance | 5.201 (.083) | 62.857 | *** | .659 |
|  |  |  |  |  |
| 2020 | Unstandardised estimate (SE) | z-value |  | Standardised estimate |
| Gender | -.750 (.076) | -9.864 | *** | -.135 |
| Age Group | -.185 (.029) | 6.262 | *** | .088 |
| Social Grade | -.098 (.077) | -1.272 | .203 | -.017 |
| Enhancement | .954 (.048) | 19.753 | *** | .367 |
| Social | .228 (.048) | 4.710 | *** | .089 |
| Conformity | -.399 (.063) | -6.339 | *** | -.099 |
| Coping | .742 (.050) | 14.813 | *** | .228 |
|  |  |  |  |  |
| AUDIT_C |  |  |  |  |
| Intercept | 2.031 (.244) | 8.334 | *** | .733 |
| Variance | 5.343 (.123) | 43.578 | *** | .695 |

****p* < .001

***Table S5. Model parameters for M2 (Regression coefficients held equal) 2018 vs 2020 data***

| 2018 | Unstandardised estimate (SE) | z-value | *p*-value | Standardised estimate |
| --- | --- | --- | --- | --- |
| Gender | -.974 (.043) | -22.676 | *** | -.174 |
| Age Group | .100 (.015) | 6.790 | *** | .056 |
| Social Grade | -.251 (.043) | -5.781 | *** | -.044 |
| Enhancement | 1.024 (.028) | 36.848 | *** | .394 |
| Social | .227 (.027) | 8.360 | *** | .089 |
| Conformity | -.530 (.037) | -14.416 | *** | -.126 |
| Coping | .672 (.029) | 23.469 | *** | .209 |
|  |  |  |  |  |
| AUDIT_C |  |  |  |  |
| Intercept | 2.823 (.137) | 20.534 | *** | 1.011 |
| Variance | 5.209 (.083) | 62.857 | *** | .668 |
|  |  |  |  |  |
| 2020 | Unstandardised estimate (SE) | z-value |  | Standardised estimate |
| Gender | -.974 (.043) | -22.676 | *** | -.173 |
| Age Group | .100 (.015) | 6.790 | *** | .047 |
| Social Grade | -.251 (.043) | -5.781 | *** | -.044 |
| Enhancement | 1.024 (.028) | 36.848 | *** | .389 |
| Social | .227 (.027) | 8.360 | *** | .088 |
| Conformity | -530 (.037) | -14.416 | *** | -.130 |
| Coping | .672 (.029) | 23.469 | *** | .204 |
|  |  |  |  |  |
| AUDIT_C |  |  |  |  |
| Intercept | 2.993 (.135) | 22.124 | *** | 1.065 |
| Variance | 5.383 (.124) | 43.578 | *** | .682 |

****p* < .001

***Table S6.* *Model parameters for M3 (Regression coefficients and intercepts held equal) 2018 vs 2020 data***

| 2018 | Unstandardised estimate (SE) | z-value | *p*-value | Standardised estimate |
| --- | --- | --- | --- | --- |
| Gender | -.975 (.043) | -22.672 | *** | -.174 |
| Age Group | .088 (.014) | 6.100 | *** | .049 |
| Social Grade | -.253 (.044) | -5.820 | *** | -.045 |
| Enhancement | 1.026 (.028) | 36.893 | *** | .394 |
| Social | .225 (.027) | 8.302 | *** | .089 |
| Conformity | -.531 (.037) | -14.461 | *** | -.126 |
| Coping | .671 (.029) | 23.422 | *** | .209 |
|  |  |  |  |  |
| AUDIT_C |  |  |  |  |
| Intercept | 2.932 (.134) | 21.834 | *** | 1.049 |
| Variance | 5.210 (.083) | 62.857 | *** | .668 |
|  |  |  |  |  |
| 2020 | Unstandardised estimate (SE) | z-value |  | Standardised estimate |
| Gender | -.975 (.043) | -22.672 | *** | -.173 |
| Age Group | .088 (.014) | 6.100 | *** | .041 |
| Social Grade | -.253 (.044) | -5.820 | *** | -.044 |
| Enhancement | 1.026 (.028) | 36.893 | *** | .389 |
| Social | .225 (.027) | 8.302 | *** | .087 |
| Conformity | -.531 (.037) | -14.461 | *** | -.130 |
| Coping | .671 (.029) | 23.422 | *** | .203 |
|  |  |  |  |  |
| AUDIT_C |  |  |  |  |
| Intercept | 2.932 (.134) | 21.834 | *** | 1.042 |
| Variance | 5.398 (.124) | 43.578 | *** | .682 |

****p* < .001

***Table S7. Model parameters for M4 (Regression coefficients, intercepts and residuals held equal). 2018 vs 2020 data***

| 2018 | Unstandardised estimate (SE) | z-value | *p*-value | Standardised estimate |
| --- | --- | --- | --- | --- |
| Gender | -.972 (.043) | -22.616 | *** | -.173 |
| Age Group | .088 (.014) | 6.128 | *** | .049 |
| Social Grade | -.252 (.044) | -5.786 | *** | -.044 |
| Enhancement | 1.025 (.028) | 36.866 | *** | .392 |
| Social | .225 (.027) | 8.300 | *** | .088 |
| Conformity | -.530 (.037) | -14.425 | *** | -.125 |
| Coping | .672 (.029) | 23.451 | *** | .208 |
|  |  |  |  |  |
| AUDIT_C |  |  |  |  |
| Intercept | 2.924 (.134) | 21.773 | *** | 1.043 |
| Variance | 5.271 (.069) | 76.485 | *** | .670 |
|  |  |  |  |  |
| 2020 | Unstandardised estimate (SE) | z-value |  | Standardised estimate |
| Gender | -.972 (.043) | -22.616 | *** | -.174 |
| Age Group | .088 (.014) | 6.128 | *** | .042 |
| Social Grade | -.252 (.044) | -5.786 | *** | -.044 |
| Enhancement | 1.025 (.028) | 36.866 | *** | .392 |
| Social | .225 (.027) | 8.300 | *** | .088 |
| Conformity | -.530 (.037) | -14.425 | *** | -.131 |
| Coping | .672 (.029) | 23.451 | *** | .205 |
|  |  |  |  |  |
| AUDIT_C |  |  |  |  |
| Intercept | 2.924 (.134) | 21.773 | *** | 1.048 |
| Variance | 5.271 (.069) | 76.485 | *** | .677 |

****p* < .001

***Table S8.* *Invariance tests (model fits and comparisons). 2018 vs 2020 data***

| Model | Χ^2^ (df) | CFI | RMSEA | SRMR | Model comp | ΔΧ2 (Δdf) | ΔCFI | ΔRMSEA | ΔSRMR | Decision |
| --- | --- | --- | --- | --- | --- | --- | --- | --- | --- | --- |
| M1: Baseline model | 0 (0) | 1.000 | .000 | .000 | - | - | - | - | - | - |
| M2: Regression coefficients equal | 7 (39.45) ** | .993 | .028 | .007 | M1 | 39.45 (7) ** | -.007 | .028 | .007 | Accept |
| M3: Intercepts equal | 8 (52.55) ** | .990 | .031 | .008 | M2 | 13.10 (1) ** | -.003 | .003 | .001 | Accept |
| M4: Residuals equal | 9 (54.17) | .990 | .029 | .007 | M3 | 1.62 (1) | .000 | -.002 | .000 | Accept |

*Note. N = 11,700; 2018 sample n = 7902; 2020 sample n = 3798.*

**p ≤ .05, **p ≤ .01*
